# Endoscopic submucosal dissection of early gastric cancer using a novel image-enhanced endoscopy: amber-red color imaging

**DOI:** 10.1055/a-2357-8351

**Published:** 2024-07-26

**Authors:** Kohei Funasaka, Noriyuki Horiguchi, Hyuga Yamada, Keishi Koyama, Teiiji Kuzuya, Ryoji Miyahara, Yoshiki Hirooka

**Affiliations:** 1 12695 Department of Gastroenterology and Hepatology, Fujita Health University, Toyoake, Japan

Hemostasis during endoscopic submucosal dissection (ESD) is essential to ensuring success with few complications 1 2 . FUJIFILM has recently developed a novel image-enhanced technology for ESD, called amber-red color imaging (ACI). ACI improves the visualization of blood flow in bleeding situations by using amber and orange colors while keeping the submucosal layer blue. We report two cases of gastric ESD using continuous ACI ( Video 1 ).

Endoscopic submucosal dissection of early gastric cancer using a novel image-enhanced endoscopy: amber-red color imaging.Video 1

Case 1: A 53-year-old man with an 8-mm type 0-IIc lesion in the greater curvature of the gastric antrum.

Case 2: A 61-year-old man with a 25-mm type 0-IIa+IIc lesion in the posterior wall of the gastric upper body.

Gastric ESD was performed using an EG-840T endoscope with a novel processor EP-8000 (FUJIFILM Co., Tokyo, Japan). After submucosal injection of indigo carmine-containing glycerol, the submucosa appeared vivid light blue and was more easily distinguished from the mucosa or muscle layers than with white-light imaging (WLI) ( Fig. 1 ). Blood flow was easily visualized once bleeding occurred, thereby allowing accurate identification of bleeding points ( Fig. 2 ). ESD was completed without any visual changes to the submucosal color.

**Fig. 1:**
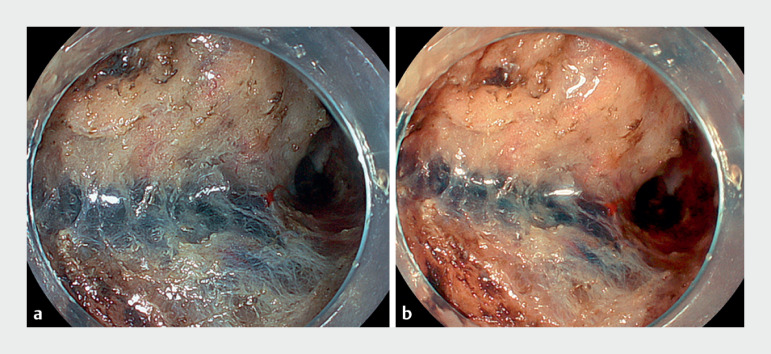
Endoscopic images of the submucosa during endoscopic submucosal dissection. **a** White-light imaging. **b** Amber-red color imaging.

**Fig. 2:**
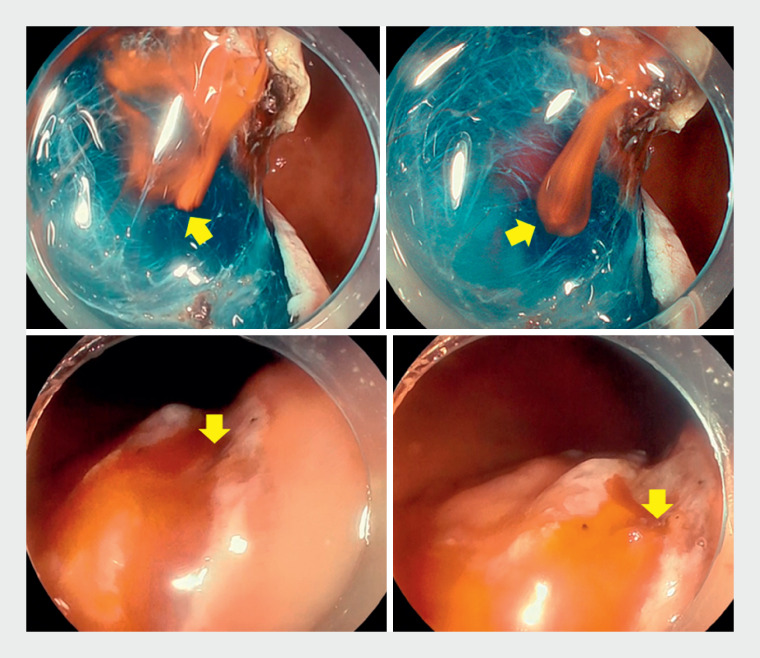
Endoscopic images of the bleeding scene with amber-red color imaging. The yellow arrows indicate bleeding points.

ACI involves the use of amber-red, green, and blue light-emitting diodes (LEDs). The spectral profile of the LED light source is controlled to make differences in the shading of blood easier to see. To ensure blood flow is easily recognized on the images, changes in brightness and hue are limited to the red color range. Moreover, ACI maintains other color tones that are similar to those of WLI. The current ACI represents an improvement of similar image-enhanced technologies using white-light illumination 3 . Red dichromatic imaging, which is similar to ACI, has been reported to provide better visualization of bleeding points 4 5 ; however, the submucosal color during ESD was different from ACI. In conclusion, the novel ACI technology would improve the efficiency of ESD.

Endoscopy_UCTN_Code_TTT_1AO_2AM

## Notes

Endoscopy E-Videos https://eref.thieme.de/e-videos
*E-Videos* is an open access online section of the journal *Endoscopy* , reporting on interesting cases and new techniques in gastroenterological endoscopy. All papers include a high-quality video and are published with a Creative Commons CC-BY license. Endoscopy E-Videos qualify for HINARI discounts and waivers and eligibility is automatically checked during the submission process. We grant 100% waivers to articles whose corresponding authors are based in Group A countries and 50% waivers to those who are based in Group B countries as classified by Research4Life (see: https://www.research4life.org/access/eligibility/ ). This section has its own submission website at https://mc.manuscriptcentral.com/e-videos .
